# External amplitude and frequency modulation of a terahertz quantum cascade laser using metamaterial/graphene devices

**DOI:** 10.1038/s41598-017-07943-w

**Published:** 2017-08-09

**Authors:** S. J. Kindness, D. S. Jessop, B. Wei, R. Wallis, V. S. Kamboj, L. Xiao, Y. Ren, P. Braeuninger-Weimer, A. I. Aria, S. Hofmann, H. E. Beere, D. A. Ritchie, R. Degl’Innocenti

**Affiliations:** 1 Cavendish Laboratory, University of Cambridge, J J Thomson Avenue, Cambridge, CB3 0HE United Kingdom; 2 Dept. of Engineering, University of Cambridge, 9 J J Thomson Avenue, Cambridge, CB3 0FA United Kingdom; 3 Purple Mountain Observatory, Chinese Academy of Sciences, 2 West Beijing Road, Nanjing, JiangSu 210008 China; 4 School of Aerospace, Transport and Manufacturing, Cranfield University, College Road, Cranfield, MK43 0AL United Kingdom

## Abstract

Active control of the amplitude and frequency of terahertz sources is an essential prerequisite for exploiting a myriad of terahertz applications in imaging, spectroscopy, and communications. Here we present a optoelectronic, external modulation technique applied to a terahertz quantum cascade laser which holds the promise of addressing a number of important challenges in this research area. A hybrid metamaterial/graphene device is implemented into an external cavity set-up allowing for optoelectronic tuning of feedback into a quantum cascade laser. We demonstrate powerful, all-electronic, control over the amplitude and frequency of the laser output. Full laser switching is performed by electrostatic gating of the metamaterial/graphene device, demonstrating a modulation depth of 100%. External control of the emission spectrum is also achieved, highlighting the flexibility of this feedback method. By taking advantage of the frequency dispersive reflectivity of the metamaterial array, different modes of the QCL output are selectively suppressed using lithographic tuning and single mode operation of the multi-mode laser is enforced. Side mode suppression is electrically modulated from ~6 dB to ~21 dB, demonstrating active, optoelectronic modulation of the laser frequency content between multi-mode and single mode operation.

## Introduction

Terahertz (THz) technologies have experienced rapid growth in recent years thanks to the enormous progress of THz sources and instrumentation. Broadband THz time domain spectroscopy (TDS) and high power semiconductor quantum cascade lasers (QCLs) have begun to unlock a myriad of THz applications in spectroscopy, imaging and communications^1^. The realization of more compact, versatile and less expensive fs-pulsed lasers has enabled the progress of TDS THz sources. QCLs, a complementary THz source, are widely used because of the wide range of available lasing frequencies and their high, stable output power and narrow frequency^2–5^. THz radiation is already being implemented for biological imaging, medical imaging, healthcare, and security. It is considered to play a dominant role in the development of high speed wireless communications as it is a non-allocated frequency range, offering transmission rates exceeding 100 Gbit/s^6^, and holds significant promise for local communications, e.g. in hospitals and universities, or in military and satellite communications. For all of these applications it is essential to have the capacity to modulate and control the amplitude, phase and frequency of radiation from the THz source and ideally gating at GHz speeds.

For the development of any future THz communications platform, it is essential to implement fast electronic control over the amplitude and frequency of the signals. An important step in this direction has been achieved recently by monolithically integrating a graphene layer onto a QCL active region for amplitude modulation^7^, achieving 100 MHz modulation speeds and an amplitude modulation depth of 100%. An alternative modulation system, allowing for the use of a standard laser source, involves reflecting the laser output beam from an external device with a reflectivity which can be electrically modulated. Graphene loaded metamaterial structures are used to build external modulators for this purpose, demonstrating fast modulation in the mid infrared region^8–11^ and also in the THz regime^12, 13^ with modulation speeds > 100 MHz being achieved^14^. Graphene is a suitable active material to be combined with metamaterials as the room temperature mobility can be as high as ~105 cm^2^V^−1^s^−1^ and the carrier concentration can be electrically tuned from very low values at the Dirac point to values of up to ~10^14^ cm^−2^ ^15^. Semiconductor two-dimensional electron gases (2DEGs) can also be used as an active material to build metamaterial devices^16^ however graphene provides a much larger tuning range^17^. Also, large scale grown CVD graphene is very easy to integrate with gold metamaterials arrays and can be easily shaped using standard etching techniques. Photo-excitable Silicon and GaAs has also been used as an active medium in metamaterial devices, e.g. in ref. 18, however this requires an excitation laser for tuning and thus requires the use of costly, extra equipment, in comparison with a device which uses a pure electronic modulation technique. Microelectromechanical systems (MEMS) have been used to physically modulate the shape of a metamaterial device^19^ giving a strong tuning range, however there is a speed limitation to such devices due to the physically moving parts and the fabrication is often highly complex.

Graphene loaded metamaterial external modulation devices^12–14^ are ideal for communications as they work at room-temperature, are all-electronic, and facilitate the separation of modulating components from the emitting devices which is essential for quadrature amplitude modulation (QAM) communication systems. However, the maximum amplitude modulation depth using this external modulator approach tends to be limited to <30%. We present a method of improving this limited amplitude modulation depth using a similar external modulator, however, implemented in an external cavity QCL (EC-QCL) configuration. The external modulator used for this experiment is built using graphene loaded metamaterial arrays which can be electrically gated to tune the reflectivity. In this configuration, the QCL emission is totally suppressed when no feedback is present by attaching a parylene coated silicon lens onto the laser facet, similar to the approach used in ref. 20. When implementing the graphene loaded metamaterial amplitude modulator as an external reflector, lasing action is restored and the output power of the QCL can be electronically manipulated by tuning the reflected feedback strength. Due to the non-linear nature of laser gain, the external cavity set-up amplifies any small changes in the reflected radiation fed back into the active region and a fast optoelectronic chopper is realized, capable of achieving 100% amplitude modulation depths around laser threshold. This set-up can be used for source amplitude modulation for high speed THz communications and could also be used as an external cavity chopper to improve the acquisition time of QCL self-mixing imaging experiments, replacing typical slow mechanical chopper methods^20–22^.

For spectroscopy purposes, QCLs are extensively used in environmental monitoring, astronomical applications^23^, gas sensing^24^, and metrology^25^. Almost all of these applications require active, fast control over the source frequency and ideally the capability to continuously sweep over 100 s of GHz. This issue has been addressed in several experiments implementing, for example, electrical tuning of the QCL^26, 27^, temperature tuning^28^ and the cavity pulling effect^29, 30^. External cavities have been used to tune the frequency by changing the length of the cavity and mechanically rotating frequency selective feedback elements^31–34^. However, for efficient spectroscopic measurements, fast frequency tuning over large sweeps is necessary, and current techniques are either slow or deliver a limited frequency modulation range (~10 GHz).

Due to the inherently dispersive nature of the reflectivity of metamaterials, it is possible to actively control the frequency content of the radiation fed back into our QCL with the metamaterial/graphene device, hence the output spectra can be electronically controlled. Frequency tuning and efficient mode suppression is demonstrated by implementing lithographic and electrical tuning of the feedback device. This approach has been used to electronically manipulate the spectral output of the QCL, converting the emission frequency content from multimode to single mode with a side mode suppression of ~21 dB. Single mode emission of a THz source is of fundamental importance for many spectroscopic applications, e.g. gas sensing. Finally, the versatility of this method could be further exploited by engineering more complex metamaterial resonances to match the required dispersive profile of QCL feedback, ideally over a large bandwidth (>1 THz). This could be implemented in THz QCL active modelocking^35–38^, similar to a layout operating in the infrared region in ref. 39. The active metamaterial/graphene feedback approach is suitable as it could potentially yield modulation speeds comparable to the cavity round trip whilst simultaneously compensating for the group delay dispersion of the active region.

## Results

**Figure 1:**
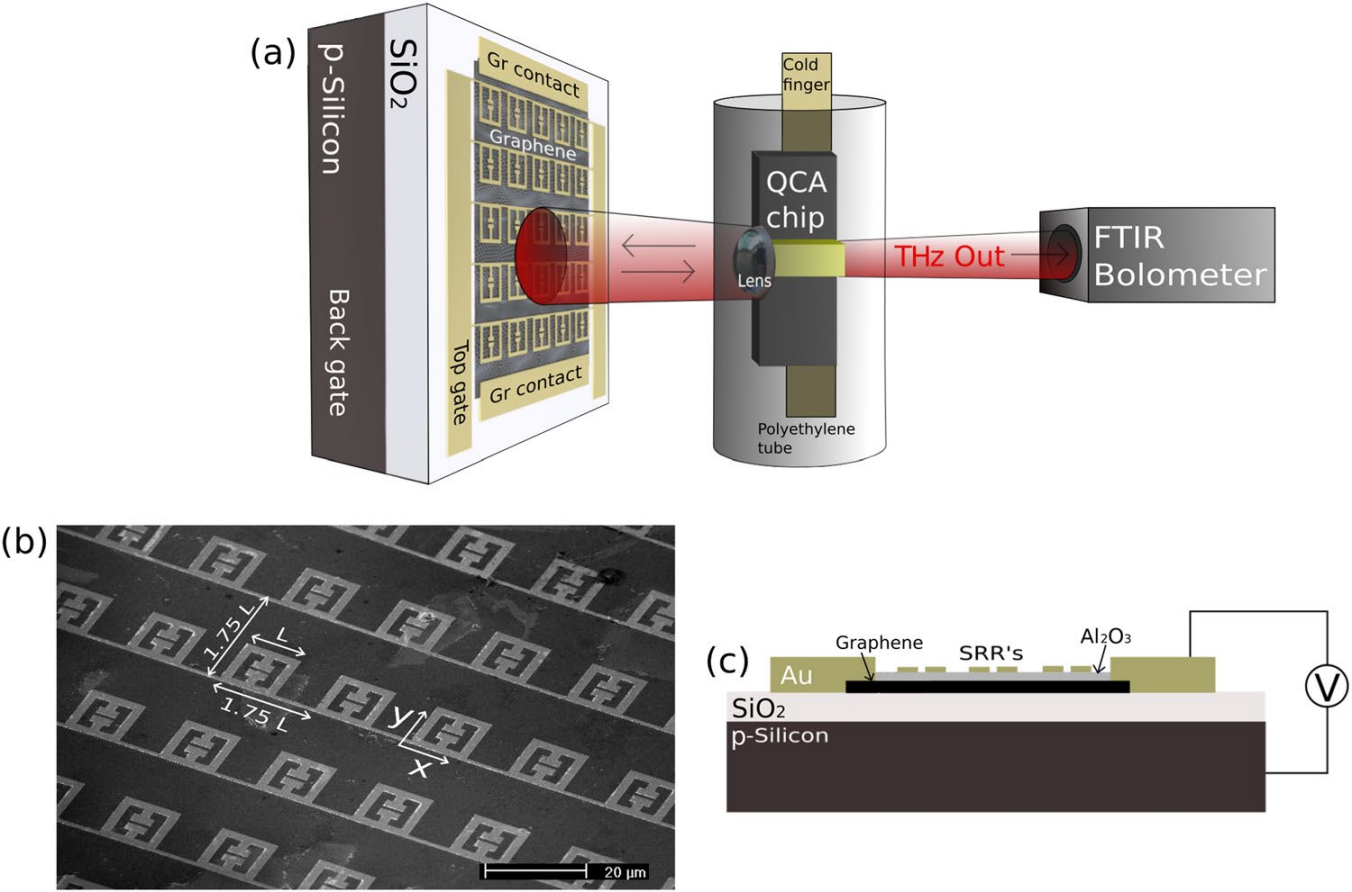
(**a**) Schematic of the set-up used for characterization of the EC-QCL. (**b**) SEM image of SRR/graphene device showing length (L) and pitch (1.75 L). (**c**) Cross sectional representation of the fabricated device illustrating the back-gating biasing scheme implemented throughout the measurements.

A metamaterial/graphene device with an electrically tunable reflection has been designed and implemented as a reflective element in an external cavity set-up for active external control of a QCL source. This set-up is built around a multi-mode bound to continuum QCL emitting at 2.9 THz^40^, fabricated in a single plasmon waveguide. Lasing is fully suppressed by applying a parylene (18.5 μm thickness) anti-reflection coated silicon lens to the back facet of the QCL using PMMA, effectively converting the QCL into a quantum cascade amplifier (QCA)^20, 41^. This QCA is mounted onto the cold-finger of a continuous flow liquid helium cryostat with a THz transparent polyethylene tube used as the outer sheath for coupling of the radiation. The reflective metamaterial/graphene feedback element is mounted onto an xyz-stage at a nominal distance of 1 cm from the polyethylene tube to complete the external cavity with this set-up schematically illustrated in Fig. 1a.

Two similar devices (device 1 and device 2) are fabricated using a split ring resonator (SRR) metamaterial structure. Device 1 is designed to have a reflectance peak at around 2.8 THz and Device 2 was designed to have 4 separate SRR areas with reflectance peaks ranging from 2.85 to 3.0 THz. A scanning electron microscope (SEM) picture of the SRR array for device 1 is shown in Fig. 1b. A p-doped substrate with a SiO_2_ insulating layer is used for these devices with a graphene layer placed on top using chemical vapor deposition (CVD)^42^ and the gold metamaterial array deposited on top of the graphene. The graphene is used to dampen the metamaterial resonances and the level of dampening can be electrically controlled by electrostatically back-gating the graphene, tuning the graphene conductivity and modulating the reflection amplitude. An encapsulation layer of Al_2_O_3_ is used to passivate the graphene using electron beam deposition for device 1 and atomic layer deposition (ALD) for device 2. The fabrication cross section along with the electrostatic back-gating method is illustrated in Fig. 1c. The experimental set-up, device simulation and fabrication is further discussed in the methods section.

### Electrical characterization

**Figure 2:**
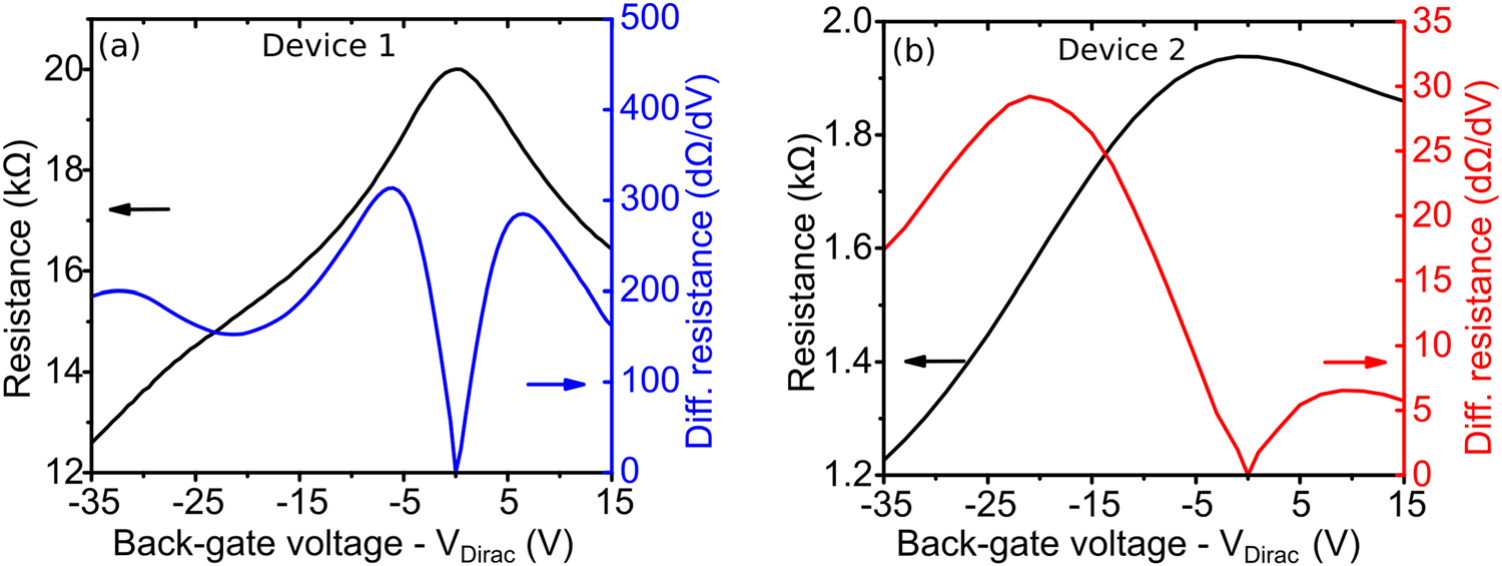
Measured graphene resistance for different back-gate voltages (black) and calculated differential of resistance with respect to back-gate voltage (blue and red). (**a**) Device 1; (**b**) Device 2.

Before performing QCA feedback measurements, the sheet conductivity of the graphene in each device was characterized at different back-gate voltages by measuring the resistance between the two graphene contacts. Two Model 2400 Keithley source/measure units (SMUs) were used with the first SMU sourcing a constant current (typically <10 μA) between the graphene contacts. The voltage required to provide this constant current was measured and the resistance determined. The second SMU is used to sweep a DC voltage applied between the p-doped Si substrate and graphene contacts, while ensuring the leakage current remains below 100 nA. The Dirac point, corresponding to the back-gate voltage of minimum graphene carrier concentration, for each device is determined from these measurements. Dirac points of 25 V and 35 V were measured for device 1 and device 2 respectively, which is closer to 0 V than the graphene Dirac point typically measured when no encapsulation layer is used with values of >100 V expected^12^. The resistance measurements for back-gate voltages from V_Dirac_ − 35 to V_Dirac_ + 15 for device 1 and device 2 are shown in Fig. 2a and b respectively. The graphene sheet conductivity range is determined to be 0.05–0.11 mS for device 1 and 0.3–1.1 mS for device 2, informing the COMSOL simulations which were used to design these devices. This allows for specific back-gate dependent reflection curves for each device to be determined (shown in SI).

The differential of the resistance vs back-gate voltage is calculated from these measurements and also presented in the figures as it is an effective way of precisely determining the Dirac point, located when the differential goes to zero. One key difference between the two figures is that the absolute value of the graphene resistance is a factor of 10 lower for device 2 with the graphene encapsulated using ALD. The stop-flow mode ALD technique is capable of uniformly depositing continuous, pinhole-free encapsulation layer on graphene^43^, resulting in higher graphene mobility. This encapsulation technique also proved to be far more effective at passivating the graphene than the electron beam deposition encapsulation technique used for device 1. The Dirac point of device 1 drifted to higher voltages by tens of volts over the course of months, whereas the Dirac point of device 2, with the graphene successfully passivated using ALD of a Al_2_O_3_ layer^44^, remained at around 35 V for the entirety of the experiment.

### Amplitude modulation

**Figure 3:**
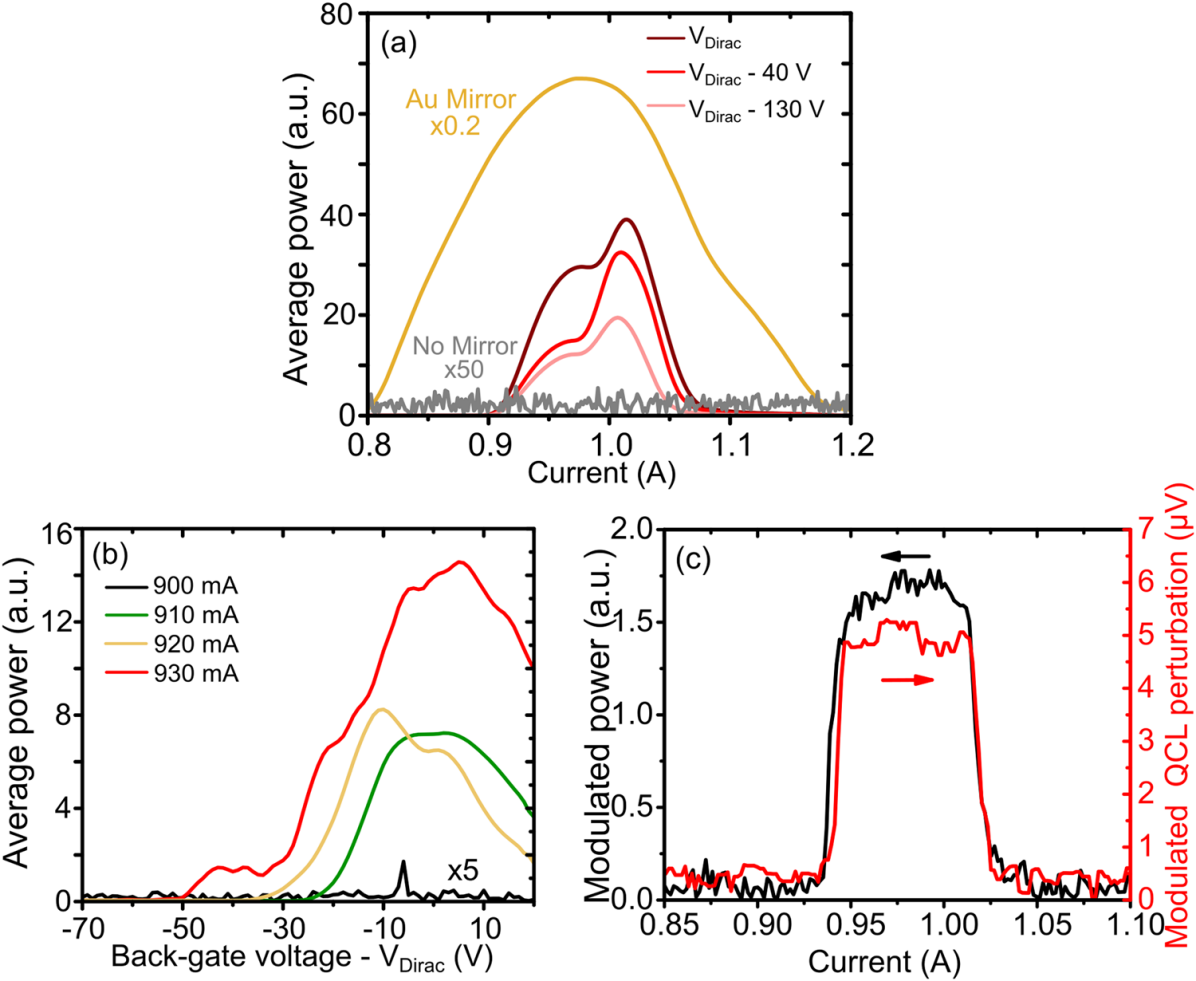
(**a**) L-I measurements when no mirror is used for feedback compared to the case when an Au mirror is used and device 1 is used for feedback at different back-gate voltages. (**b**) Power output around threshold current when device 1 is used for feedback and set to different back-gate voltages. (**c**) Modulated power from the EC-QCL when a square wave voltage is applied to the back-gate of device 1 from V_Dirac_ − 60 V to V_Dirac_ − 40 V. The laser output power perturbation as measured by a bolometer (black curve) and perturbation voltage simultaneously measured across the QCA (red curve).

Before the SRR/graphene devices were implemented into the QCA feedback set-up a standard gold mirror (reflectivity 99.7%) was used as the feedback element converting the QCA into an EC-QCL. A characterization of the EC-QCL output power across a full range of currents was performed and is presented in Fig. 3a. With no external mirror present, there is no output power measured above the noise floor due to the anti-reflection coated lens, demonstrating the successful suppression of the QCL. With the gold mirror placed 1 cm in front of the lens for feedback, lasing is observed for QCA currents ranging from 800 to 1200 mA. The threshold current and laser power are strongly dependent on the strength of feedback into the QCA with both the dynamic range and maximum average power decreasing as the feedback strength is reduced.

The feedback percentage of the external cavity mirror into the QCA active region can be estimated by comparing the threshold current in this configuration with the threshold current of the QCL when no suppression lens is attached and no external feedback is present. Using a method described in ref. 34 the feedback strength from the gold mirror can be determined by assuming the ratio of the threshold currents is proportional to the ratio of the total roundtrip losses in these configurations. The QCL threshold with no suppression and no external feedback was measured to be 700 mA and assuming the QCL facet has a reflectivity of 32% with a waveguide loss of 7 cm^−1^ the percentage feedback into the lens side of the QCA from the external cavity mirror system is estimated to be 13% using the new threshold value of 800 mA. This is low due to the alignment of the lens and mirror system and could be optimized further to increase this feedback percentage.

In order to achieve active control of the EC-QCL device 1 replaced the Au mirror as the reflecting element and the output power was characterized for a range of different device back-gate voltages. Figure 3a shows that when using this SRR device as the external cavity mirror, a higher threshold current (~900 mA) is observed and a smaller dynamic range (~150 mA) is available compared to when the Au mirror was used. Using the same threshold current ratio method as with the mirror feedback, the peak SRR device feedback percentage is estimated to be 5%. This change is partially explained by the fact that the SRR reflectivity at 2.9 THz is around 70% compared to the mirror which is around 99.7%. This difference in reflected power also explains why the output power is 9 times lower.

When the back-gate of device 1 is reduced away from V_Dirac_, the reflectivity at 2.9 THz is predicted by the COMSOL simulation (in SI) to reduce by up to 15%. Due to this change in reflectivity, the feedback strength and hence the peak output power from the QCL is electronically modulated. From Fig. 3a the output power of the EC-QCL increases as the back-gate voltage moves towards V_Dirac_, leading to a modulation depth of around 55% at I_max_ = 1000 mA, observed when sweeping the back-gate from V_Dirac_ to V_Dirac_ − 130 V. Two peaks in the output power can be seen on either side of a dip at around 980 mA which would indicate mode-hopping. However, the laser is far more sensitive to feedback changes at the threshold current, I_threshold_, and this sensitivity can be taken advantage of to increase the modulation depth.

If the QCL is operated just below threshold, with the SRR/graphene device operating at low reflectivity (e.g. V_Dirac_ − 70 V), the QCL will be in a superluminescence/non-lasing state giving essentially zero output power. The output power for different QCL currents is shown in Fig. 3b with the voltage across device 1 swept from V_Dirac_ − 70 V to V_Dirac_ + 20 V. As the feedback is increased, the threshold current of the EC-QCL system switches from a non-lasing state to a lasing state. Due to the sensitivity of this threshold condition, modulation depths of 100% are achieved as shown in Fig. 3b. The turn on back-gate voltage, when the QCL switches from non-lasing to lasing, is dependent on the QCL operating current with lower currents requiring the voltage to be closer to V_Dirac_ before lasing can be achieved. The maximum power is obtained when the back-gate voltage is near V_Dirac_. There is a slight offset of this peak of around 10 V for the measurements at 900 and 920 mA which is most likely due to mechanical instability of the external cavity system causing variations in the output power. This could also be due a variation in the position of V_Dirac_ due to graphene hysteresis or doping from the environment, highlighting the importance of the ALD encapsulation process^44^. This demonstrates the ability to switch the QCL on and off with modulation depths of 100% using an external optoelectronic switch. Also, if our devices were configured for high speed modulation by reducing the size of the metamaterial array and the surface area of the graphene, the capacitance of the device could be reduced. This would allow us to switch the laser on and off at modulation speeds of >100 MHz. These modulation speeds are demonstrated by one of our previous metamaterial/graphene amplitude modulation devices reported in ref. 14.

To illustrate the external chopper capabilities of the SRR/graphene feedback, a 330 Hz, 20 V peak to peak square wave centered around V_Dirac_ − 50 V was applied to the back-gate of device 1 and the change in output power of the EC-QCL was measured. In Fig. 3c the modulation depth from this back-gate 20 V square wave is measured at different QCA operating currents from 850 mA to 1100 mA using a lock-in amplifier with a reference frequency of 330 Hz to demodulate the chopper signal. A constant modulation in the output power across the full dynamic range of the EC-QCL is measured, despite the fact that the average output power will change as the current is swept. Figure 3c also shows the simultaneously measured voltage perturbation across the QCA using a measurement technique similar to one described in ref. 20 with a 330 Hz band pass filter added. The voltage perturbation across the QCA follows the same functional form as the output power, confirming the result.

**Figure 4:**
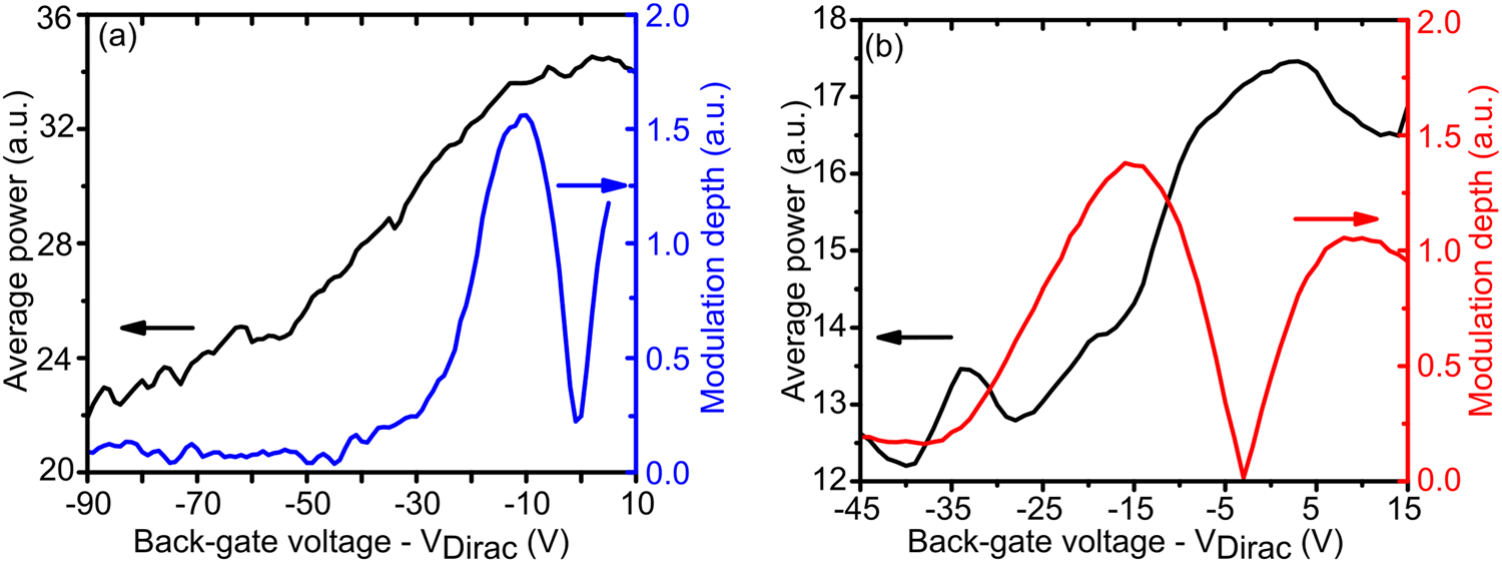
QCA average power output vs back-gate voltage of feedback device (black), modulation depth with 10 V back-gate modulation vs set point voltage (blue and red curves). (**a**) Device 1 (L = 12 μm), (**b**) Device 2 (L = 12.9 μm).

QCL amplitude modulation was also performed when implementing device 2 as a feedback element instead of device 1. EC-QCL output power values, with the QCA operating at I_max_, are reported as a function of the back-gate voltage for both devices in Fig. 4, together with the differential modulation depth recorded with a 10 V peak to peak square wave modulation added to the back-gate set-point. Figure 4 shows a modulation depth of around 35% for device 1 and a modulation depth of around 31% for device 2 for a limited range of back-gate voltages, with the QCA running at I_max_. This average power output follows the same trend as the graphene resistance measurements in Fig. 2 as the device back-gate voltage is changed. The EC-QCL output power and graphene resistance are therefore closely linked quantities which is expected as the reflectivity is dampened by an increase in graphene conductivity.

Two independently fabricated external optoelectronic amplitude modulators have been successfully implemented in a EC-QCL system, enhancing the amplitude modulation depth of these devices. By only modulating the reflectivity strength of the SRR devices by a maximum of 15–20%, EC-QCL output power modulation depths up to 55% are observed at I_max_ with 100% observed near I_threshold_ due to the intrinsic non-linear nature of the laser cavity gain.

### Frequency control

Since these devices have a non-uniform reflectivity in the frequency domain they will influence the spectrum of the laser by feeding back more photons near the reflectance peak of the metamaterial. When two modes are lasing, they will be competing for the gain of the active medium and hence there will be mode competition^45, 46^. If one of these laser modes is closer to the resonance frequency of the feedback device than the other, and the resonance of the peak of the SRR device is increased, more photons will be fed back at this frequency and hence it can saturate the gain for the other mode which receives less fed back photons. The dispersive feedback from the device is essentially enhancing lasing of modes near the reflection frequency peak and suppressing modes which are further away in a similar way to other frequency selective feedback elements like gratings^32^. The Q factor of the selectivity is broader in our case; however, this Q factor can be electronically modulated by varying the graphene conductivity, and hence the proportional difference between the feedback strengths of competing modes can be electrically modulated. If the device is biased away from V_Dirac_ the reflection peak will be relatively flat resulting in similar feedback strengths for a mode near the resonance and a mode further away, however if the device is biased at V_Dirac_ the Q factor of the resonance will increase and the difference in reflectivity between these modes will be magnified. Thus, the mode closer to the device reflectivity peak will be enhanced and will begin to saturate the gain for other mode causing mode suppression and potentially single mode operation of the laser. The device can therefore be used to optoelectronically tune the mode configuration of the device from multimode operation to single mode operation.

**Figure 5:**
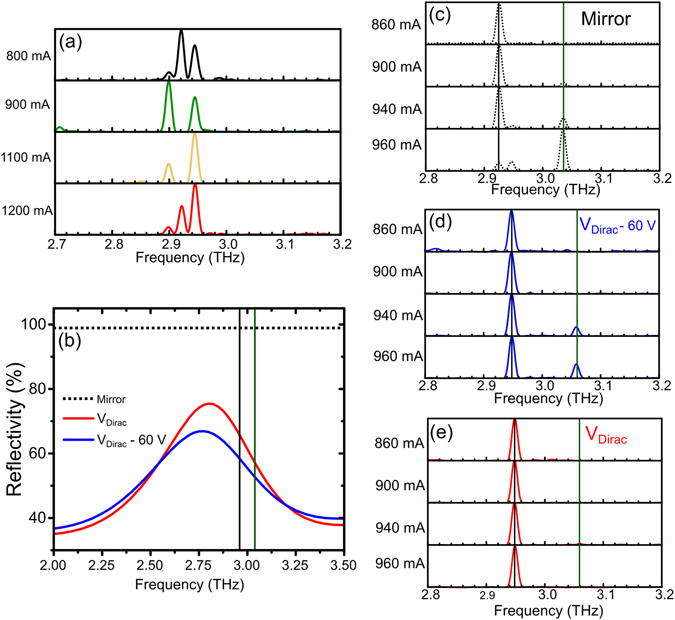
(**a**) Frequency output of Fabry-Pérot QCL with no lens attached and no feedback for different operating currents. (**b**) Simulated reflectivity of SRR at two graphene back-gate voltages and representation of flat Au mirror reflection (dotted line). (**c**–**e**) FTIR measurement of frequency output of EC-QCL at different operating currents with lens attached and feedback from mirror and device 1 (L = 12 μm) at different back-gate voltages.

The multi-mode characteristics of the QCL in standard operation, with no anti-reflection coated lens attached are shown in Fig. 5a. A clear multi-mode output consisting of 3 modes is seen with mode hopping observed throughout the QCA’s dynamic range. The spacing between these modes is determined by the QCL length and refractive index which have values of around 3 mm and n = 3.6 respectively, resulting in a free spectral range (FSR) of ~14 GHz. When the antireflection coated lens is attached the laser will be suppressed, however, the reflectivity back into the laser cavity is not completely zero (2–3%). The set-up therefore can be represented by a 3-mirror system with two cavities; one between the reflective device and the lens and another represented by the internal cavity of the QCA itself. The lasing modes in the EC-QCL set-up must align closely with the length dependent Fabry-Pérot modes of both the QCA cavity and the external cavity so that the reflected light from the external mirror can constructively interfere with the electric field inside the QCA. The operating frequency of the laser must be close to an integer number of the FSR of both the QCA and the external cavity. In this case the FSR of the QCA cavity with lens attached is estimated to be ~7 GHz and the FSR of the 1 cm external cavity is ~15 GHz. These increased frequency constraints result in 3 modes of the conventional Fabry-Pérot QCL being converted into a 2 mode laser with only two frequencies across the gain bandwidth of the laser being close enough to satisfying the external and internal cavity Fabry-Pérot conditions. The spectrum from multiple cavity lasers is discussed in detail for coupled cavity designs with two cavities on one chip^45, 47, 48^, and for external cavity feedback for diode lasers in the Lang-Kobayashi model^49^.

The Lang-Kobyashi model cannot fully describe our system however as it describes the perturbation in semiconductor lasers due to a small laser feedback: the so-called self-mixing regime^50^. Since the QCL is converted to a QCA in our system with suppressed lasing, when the feedback is added to our system it cannot be considered as a small perturbation and hence our EC-QCL is in a different external cavity regime. A full development of a theory describing the laser cavity system of this arrangement is of great interest. A further set of measurements is required in order to build a database capable of supporting a robust theoretical framework. This is, however, beyond the scope of the current manuscript.

As the current across the QCA increases, the laser mode hops to higher frequencies. If, however, the SRRs are lithographically defined such as to preferentially reflect low frequency modes, it is possible to inhibit the higher order modes from propagating, maintaining single mode operation. The simulated reflectivity of device 1 centered around 2.8 THz is shown in Fig. 5b for the graphene biased at V_Dirac_ and also at V_Dirac_ − 60 V. In this graph the reflection spectra of a standard mirror, which is flat at around 99.7% reflectivity, is shown for completeness. In all of these measurements the distance between the mirror/SRR device to the QCA lens has been set to ~1 cm. The spectral output of the EC-QCL when the flat mirror is used for feedback is shown in Fig. 5c with the output mode hopping at I_QCL_ = 960 mA from 2.95 to 3.05 THz. When device 1 is used to feedback into the QCA with the back-gate voltage set to V_Dirac_ − 60 V, less power is transferred between the modes at 960 mA. This is because, the 2.95 THz mode is preferentially reflected over the 3.05 THz mode with a 59% simulated reflectivity compared to 52%. As the back-gate voltage is increased to V_Dirac_, this selectivity becomes stronger. When the back-gate voltage is set to V_Dirac_ there is a 67% reflection simulated at 2.95 THz compared to a reflection of 56% at 3.05 THz, transferring even more power into the lower frequency mode due to mode competition. In Fig. 5e the higher frequency mode is fully suppressed at V_Dirac_ and no mode hopping is observed as the QCA current increases. This demonstrates that the SRR/graphene devices can be used to manipulate the frequency content of the EC-QCL output such as to actively suppress mode hopping. A multimode output has been converted to a single mode output by applying a bias across an external device, modulating the lateral suppression at I_QCL_ = 960 mA from 5 dB at V_Dirac_ − 60 V to 21 dB at V_Dirac_.

**Figure 6:**
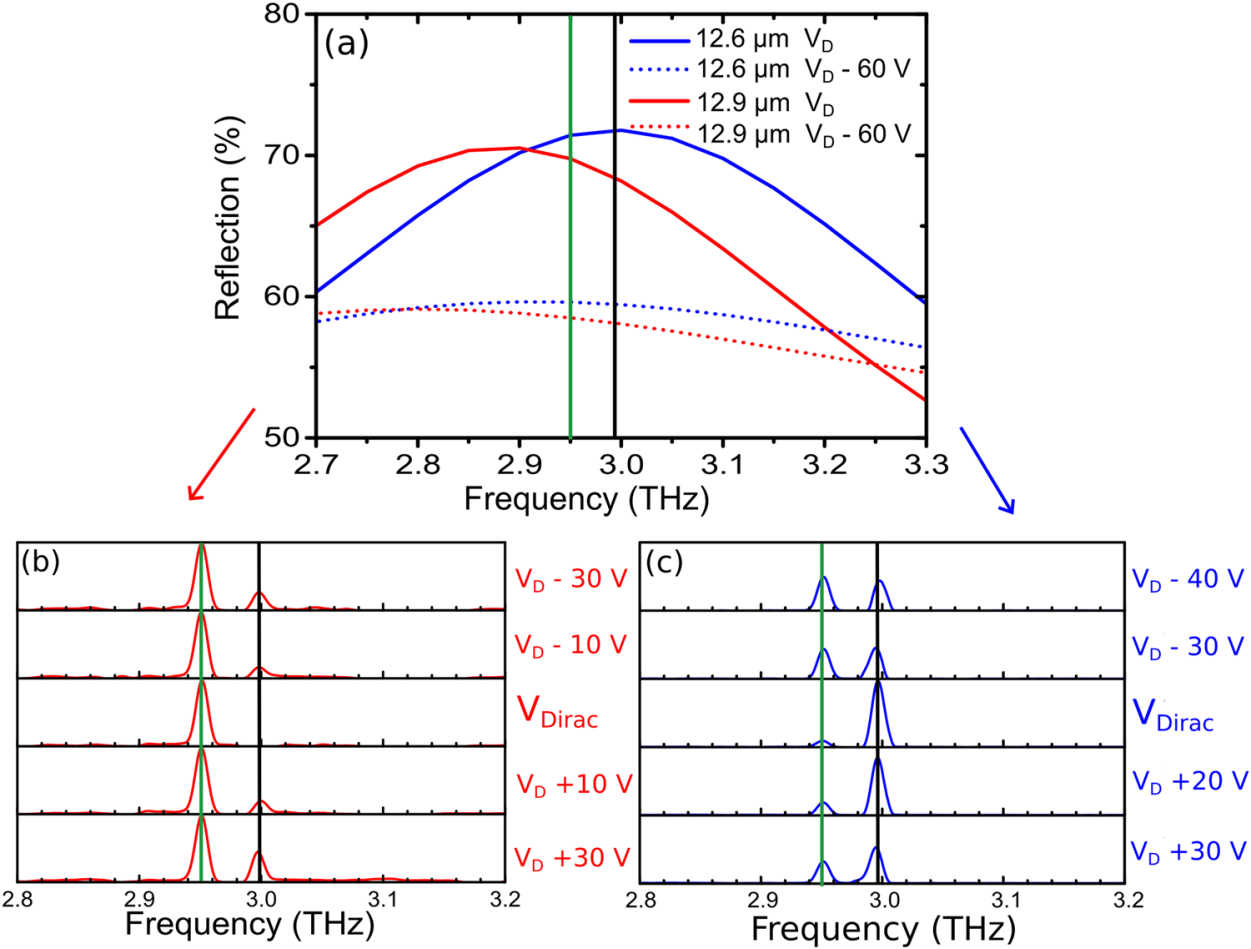
(**a**) Reflection simulation of device 2 for two SRR lengths (blue-12.6 μm, red-12.9 μm) and two back-gate voltages. (**b**,**c**) FTIR measurement of EC-QCL frequency when 12.9 μm array (red) and 12.6 μm array (blue) is used for EC feedback. Different voltages are applied to device 2 back-gate (which has a Dirac point at ~35 V) as the QCA is kept at a constant current.

To illustrate this frequency tuning further, device 2 was used for feedback, which has 4 areas containing different sizes of SRRs, exhibiting reflection peaks at different frequencies (2.85, 2.90, 2.95 and 3.0 THz). This device has two frequency tuning mechanisms which can now be taken advantage of when manipulating the spectral output of the EC-QCL. Similarly to when device 1 was used for feedback, the Q factor of the feedback selectivity can be modified by optoelectronically tuning the graphene conductivity, switching the EC-QCL output between single mode and multi-mode operation by influencing mode competition. We can however also take advantage of the 4 different lithographically tuned areas by laterally moving the device and choosing which area to independently reflect from which is possible as each area is larger than the QCA spot size as discussed in SI. The precise lithographic tuning parameters and resultant peak frequencies is discussed in the SI. The two extreme cases L = 12.9 μm and L = 12.6 μm with resonance peaks at ~2.85 THz and ~3 THz respectively are used to illustrate how the lithographic tuning of the external cavity feedback device can select which mode to enhance as the Q factor of the resonance is increased. The simulated reflectivities for these SRR areas are shown in Fig. 6a at V_Dirac_ and at V_Dirac_ − 60 V.

Device 2 is inserted into the EC-QCL set-up with the current across the QCA set to ~890 mA emitting two modes (2.95 THz and 2.99 THz). The spectrum of the laser in Fig. 6 is different compared to Fig. 3 when device 1 was used for feedback. This is because the permitted lasing modes are very sensitively dependent on the external cavity length and it is technically challenging to have the same precise length for each experiment. Hence this will result in different spectral outputs for each of the external cavity QCL configurations. Figure 6b reports the spectra when the larger size SRR area (L = 12.9 μm) is used for feedback. This figure shows the 2.95 THz peak increasing in amplitude as the Q factor of the SRR resonance increases at V_Dirac_ while the 2.99 THz peak is suppressed. As the voltage across the SRR device approaches V_Dirac_ the proportional difference between the photons fed back at 2.95 THz and 2.99 THz will increase, influencing the mode competition and enforcing single mode operation at 2.95 THz. The reverse scenario happens when the SRR device is laterally moved to reflect from the smaller SRR area (L = 12.6 μm) with a resonance peak at ~3 THz. The frequency output from the EC-QCL in this configuration is shown in Fig. 6c. As the voltage on the back-gate is moved towards V_Dirac_, the 2.99 THz peak is now enhanced while the 2.95 THz peak is suppressed showing lithographic tuning of the SRR device can be used to select which mode to optoelectronically enhance. In both cases, when the device 1 is biased above V_Dirac_ the EC-QCL goes back to multi-mode emission with the reflectance peak of the device flatting out, reducing the extent of frequency selectivity.

These SRR/graphene devices have demonstrated control over the amplitude and spectral makeup of a QCL. Lithographically selective enhancement of two different laser modes has been demonstrated using a single SRR/graphene device. The strength of the external cavity feedback tuning technique is emphasized by the very small difference in reflected percentage required for complete mode suppression. Figure 6 illustrates this strength with two low Q-factor SRR reflective devices being capable of fully isolating two peaks only separated by around 50 GHz. The difference in reflectivity between these two modes is only a few percent at V_Dirac_ for the two SRR areas and full suppression was still achieved, highlighting once more the efficiency of this feedback approach.

## Discussion

In this paper, optoelectronic external cavity control of a QCL emitting at 2.9 THz was demonstrated. Hybrid SRR/graphene based devices were used in an external cavity QCL configuration to amplify the amplitude modulation effect of these high speed devices, demonstrating an amplitude modulation depth of 100%. These devices could be used as very fast external choppers for high speed QCA imaging experiments and are also suitable for active feedback stabilization of a QCL source power. Active mode-locking of THz sources could be performed by employing this fast amplitude modulation feedback method to produce ultra-fast THz pulses. This approach also demonstrated the capability to efficiently and actively select and favor different laser modes. Electrical control of the number of modes propagating in the cavity is achieved without implementing any mechanical frequency selective elements; a tool which is of fundamental importance in THz spectroscopy. Mode hopping is inhibited by taking advantage of the frequency selective reflection of SRR’s, enforcing single mode operation of a THz source; a standard requirement for many THz applications. Strong modulation effects have been achieved by enhancing very small variations in the device reflectivity, demonstrating the strength of this non-linear external cavity feedback method for the manipulation and control of THz QCL’s. Finally, these results pave the way to the fundamental investigation of the laser dynamics in external cavity QCLs beyond the Lang-Kobayashi theory, and open new scenarios for the realization of sub-ps mode-locked QCLs.

## Methods

The metamaterial and graphene devices are built using a standard three arm split-ring resonator (SRR) array design coupled with graphene, resulting in a mirror elements with a reflectivity dependent on the graphene conductivity, similar to the amplitude modulator scheme presented in ref. 13. Two separate SRR/graphene devices labeled device 1 and device 2 are fabricated with slightly different approaches. They are both built on a boron doped p-Si substrate with a 300 nm thermally grown SiO_2_ insulating layer on top. Chemical vapor deposition (CVD) graphene^42^ is transferred onto the substrate and patterned into 3 × 3 mm^2^ squares using photo-lithography and O^2^ plasma etching. These squares are electrically contacted by source and drain pads which are defined using photo-lithography, followed by thermal evaporation of Ti/Au (10 nm/100 nm). An 80 nm insulating layer is deposited onto the graphene, however the method of deposition is different for each device. For device 1, electron beam evaporation is used to deposit Al_2_O_3_ while for device 2, atomic layer deposition (ALD) under stop-flow mode is used^44^. These two techniques result in different electrical characteristics of the graphene already discussed. For both devices, electron beam lithography is used to pattern the repeating SRR structure with Ti/Au (10 nm/90 nm) again evaporated to build the SRRs. The SRRs are electrically connected by parallel Ti/Au lines in the x direction to separate electrodes. These lines have minimal interaction with polarized radiation in the y direction and allow the SRR’s to be electrically biased as a top-gate^13^. The graphene can also be back-gated by applying a bias between the p-doped silicon substrate and the graphene contacts, and in this experiment the back-gating technique has exclusively been used as it proved to be more resilient to dielectric breakdown over months of operation.

For precise lithographic tuning of the SRR sizes, the reflectivity was modeled by a finite element method (FEM) software, COMSOL Multiphysics v 5.1 which is discussed in the SI. For device 1, the SRR length of 12 μm was designed for a reflectivity peak at around 2.8 THz. For device 2, the SRR architecture is the same, however 4 array areas were defined by different SRR lengths, L (12.6 μm, 12.7 μm, 12.8 μm and 12.9 μm) exhibiting resonance peaks at different frequencies ranging from 2.85 to 3.0 THz. A detailed characterization of the SRR parameters for each device is given in the SI.

To ensure there was no systematic error in the simulation method an SRR/graphene device was built with a simulated reflectivity at 2.2 THz which could be spectrally characterized using a THz-TDS spectrometer from Menlosystems (model Tera-K15), ensuring the resonance frequency matched simulations. The graphene conductivity range was also tested by altering the back-gate voltage and comparing the optical results with the simulations using different conductivities. These results are reported in the SI. This graphene conductivity information was used to tune the gap size of the SRR’s in order to give maximum modulation range for the available conductivities.

The lens used to convert the QCL into a QCA is a hyper-hemispherical Si lens with a diameter of 4 mm and an extension length of 2.67 mm and is also used to improve the coupling of the feedback into the QCA. The 18.5 µm thick parylene coating limits the reflection from the back facet of the QCL to <3%, fully suppressing lasing action over the QCL bandwidth. This will lead to a spot size on the feedback element of around 1.2 mm (shown in SI). The power from the emission facet of the QCA is measured using a Si-Ge 4 K bolometer. The current across the QCA is pulsed using 10 kHz square waves with a 10% duty cycle and then gated at 330 Hz to facilitate high signal to noise lock-in demodulation of the measured laser power. For spectral measurements, the output from the EC-QCL is collected by a commercial FTIR (Bruker IFS 66 v/S).

### Data availability

Additional data sets related to this publication are available from the Cambridge University data repository at https://doi.org/10.17863/CAM.11159.

## Electronic supplementary material

Supplementary Information
