# Supplementary material for: External amplitude and frequency modulation of a terahertz quantum cascade laser using metamaterial/graphene devices
Source: Sci Rep. 2017 Aug 9;7:7657. doi: 10.1038/s41598-017-07943-w (PMC5550467; doi:10.1038/s41598-017-07943-w)
Supplement: Supplementary file 1 — Supplementary Information [file 41598_2017_7943_MOESM1_ESM.pdf]

Supplementary material for "External amplitude and frequency modulation of a terahertz quantum cascade laser using metamaterial/graphene devices."

S. J. Kindness[1,†], D. S. Jessop[1], B. Wei[1], R. Wallis[1], V. S. Kamboj[1], L. Xiao[1], Y. Ren[1,*], P. Braeuninger-Weimer[2], A. I. Aria[2,**], S. Hofmann[2], H.E. Beere[1], D. A. Ritchie[1], and R. Degl'lnnocenti[1]

[1] Cavendish Laboratory, University of Cambridge, JJ Thomson Avenue, Cambridge, CB3 0HE, United Kingdom
[2] Dept. of Engineering, University of Cambridge, 9 JJ Thomson Avenue, Cambridge, CB3 0FA, United Kingdom

†sjk80@cam.ac.uk

Contents

1. FEM COMSOL Simulations……………………………………………………………2
2. TDS measurement of 2.2 THz device………………………………………………4
3. Feedback spot size…………………………………………………………………… 5
4. Feedback with graphene device……………………………………………………6
References………………………………………………………………………………7

* Current address: Purple Mountain Observatory, Chinese Academy of Sciences, 2 West Beijing Road, Nanjing, JiangSu 210008, China

** Current address: School of Aerospace, Transport and Manufacturing, Cranfield University, College Road, Cranfield, MK43 0AL, United Kingdom

1. FEM COMSOL simulations

To predict and design the reflectivity of the split ring resonator (SRR) and graphene devices we used an electromagnetic finite element method (FEM) software; COMSOL Multiphysics. In order to simulate the SRR metamaterial arrays, a unit cell is defined as shown in Fig. 1SI a) with periodic boundary conditions applied on the sides to simulate the effect of the whole array. The dimensions of the square SRR unit cell, depending on side length 'L', are also shown in this figure. For device 1, L=12 µm, Au width = 1 µm and Cgap = 1.2 µm. For device 2 there are 4 different (1.7 mm x 1.7 mm) SRR array areas. Each area has a basic SRR unit cell as shown in Fig 1SI: (a). However, for these areas to exhibit resonance peaks at different frequencies they are scaled slightly differently with four different values for the parameter L (12.6, 12.7, 12.8 and 12.9 µm) as shown in Fig. 1SI b). The position of the reflectivity peaks for these areas are simulated to be 3.0, 2.95, 2.9, 2.85 THz corresponding to the 4 different values of L in order. The LC tuning was slightly different with Cgap = 0.8 µm and with a larger Au width =2 µm which resulted in a blue shift of the resonance frequency compared to the smaller Au width in device 1. To simulate incoming THz radiation a port emitting plane waves with polarization in the y direction is defined above the SRR structure. The S_{11} and S_{21} parameters are acquired to determine the reflectivity, $|S_{11}|^2$, and the transmission, $|S_{21}|^2$. The permittivity for air, Al_2O_3 and SiO_2 were set to 1.0 and 9.1 and 3.9 respectively. The permittivity for the p-doped silicon was determined by the Drude free-carrier expression for semiconductor materials as given by equation 1[1].

$$\varepsilon_r(f) = \varepsilon_\infty - \frac{ne^2}{\varepsilon_0 m\pi(f^2 + i f\tau^{-1})} \qquad (1)$$

ε_∞ is the high frequency permittivity, n is the free charge carrier density, e is the elementary charge, ε_0 is the vacuum permittivity, f is the frequency of the electromagnetic radiation, m is the effective charge carrier mass and τ is the average lifetime of the carrier. For the p-doped silicon, values of n ~ $1.3\text{x}10^{14}$ cm^{-3} and $\tau \sim 10$ ps were used. For gold and graphene, the Drude model conductivity was first calculated from equation 2 and used in equation 3 to determine the complex permittivity.

$$\sigma(f) = \frac{\sigma_0}{(1 + i2\pi f\tau)} \qquad (2)$$

$$\varepsilon_r(f) = 1 - \frac{i\,\sigma(f)}{2\pi f \varepsilon_0} \qquad (3)$$

σ_0 is the DC conductivity which is given as $2.7\text{x}10^7$ S/m for gold and for the graphene we use a sheet

resistivity range between 0.3 and 1.1 mS for device 2 and between 0.05 and 0.11 mS for device 1, values which were obtained by measuring the graphene resistance at different back-gate voltages. τ is the average scattering time in the Drude model and values of 15 fs and 200 fs are given for graphene and gold respectively, taken from literature [2,3]. In the 3D COMSOL model, the graphene thickness was set to 15 nm and the conductivity scaled accordingly. COMSOL reflectivity simulations for device 1 and device 2 at the measured graphene conductivity extremes are shown in Fig. 1SI c) and Fig. 1SI d).

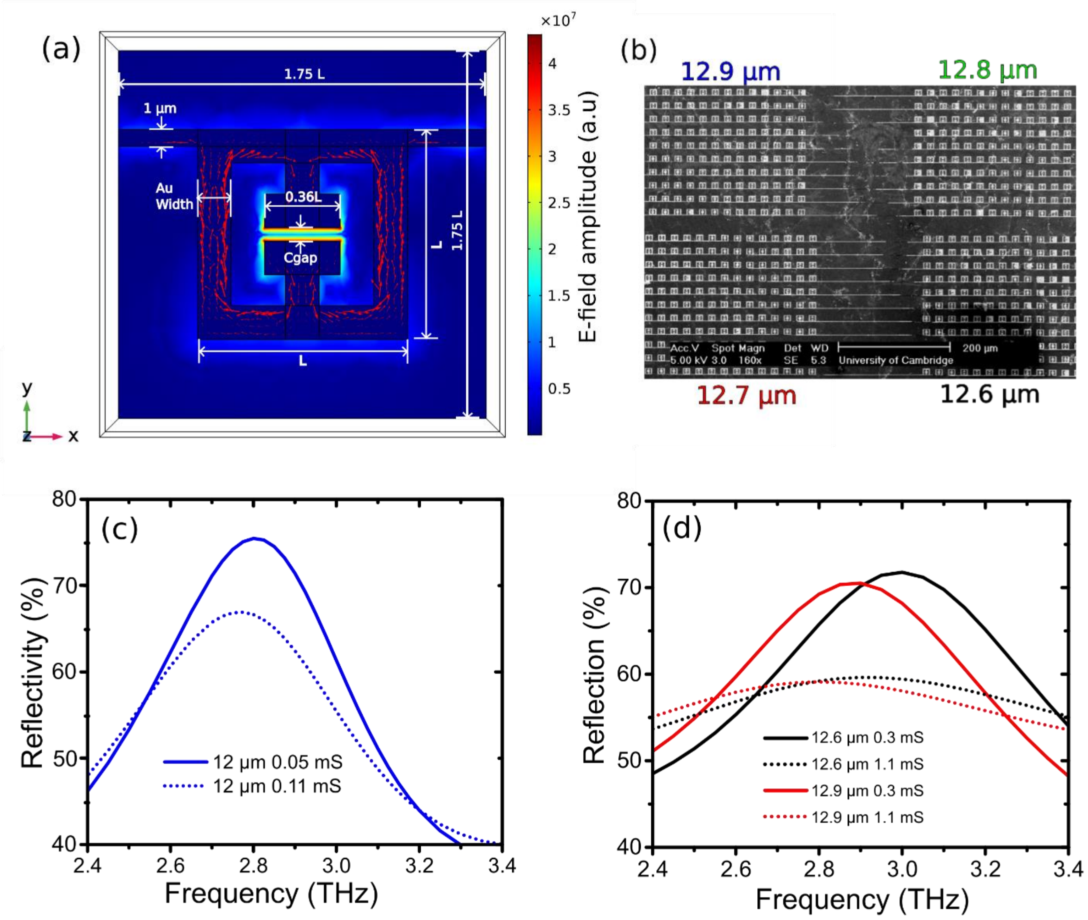

Fig 1SI: (a) COMSOL simulation showing a unit cell with dimensions as well as the E-field strength at resonance with the induced current illustrated with the red arrows. (b) SEM image of device 2 showing different reflective areas with different SRR length, L. (c) Simulation of device 1 reflectivity with graphene sheet conductivity at 0.05 and 0.11 mS. (d) Simulation of device 2 reflectivity with graphene sheet conductivity at 0.3 and 1.1 mS for L=12.6 µm and L=12.9 µm areas.

2. TDS measurement of 2.2 THz device

A THz-TDS Menlo system spectrometer model K15 was used to measure the frequency response of the 2.2 THz SRR/graphene device, characterising the resonant frequency of the metamaterial. The Menlo system spectrometer is capable of efficiently retrieving the spectral content of the object of study up to about 2.8 THz so to inform the COMSOL simulations performed for devices at 2.9 THz a scaled device was fabricated with an SRR length of 15 μm which is simulated to create a resonant frequency at around 2.2 THz. A TDS background measurement was performed for this 15 μm device with the sample rotated by 90 degrees so the SRR LC resonance was not electrically excited. The sample was now rotated to its correct polarisation and two transmission spectra, with the back-gate voltage set to V_{Dirac} and V_{Dirac} + 30 V, were taken and normalised relative to the background measurement. The results for the TDS measurement are shown in Fig. 2SI and are compared with the corresponding COMSOL simulations with graphene sheet conductivities of 0.3 mS and 0.5 mS. TDS measurements and the COMSOL simulations agree very well and hence they help to solidify our trust in the COMSOL simulation process for the 2.9 THz resonance device used in the QCA feedback experiment.

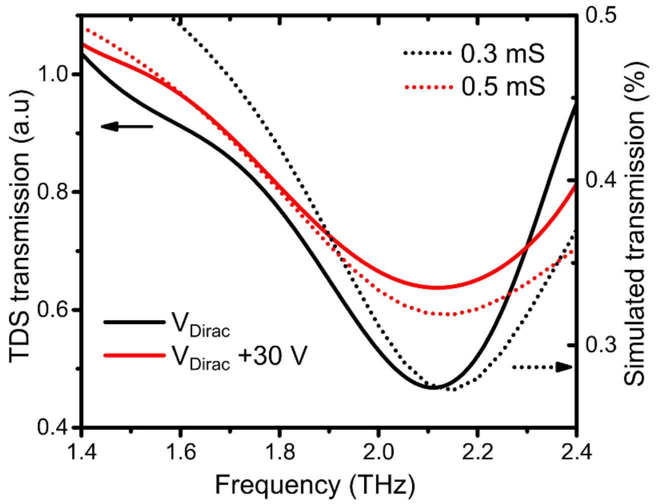

Fig 2SI: TDS transmission measurement of L=15 μm device *with reflection peak at 2.2 THz biased at different back-gate voltages (solid curves). COMSOL simulation of transmission spectrum of 2.2 THz SRR device (dotted lines) with different graphene conductivity values of 0.3 mS (black) and 0.5 mS (red).*

3. QCA feedback spot size

The spot size from the feedback lens at a distance of 1 cm is of critical importance as it determines the area of the SRR/graphene device which will feed back terahertz radiation into the QCA. The hyper-hemispherical silicon lens used in the experiment has a diameter of 4 mm and an extension length of 2.67 mm. This lens is used to improve the feedback collection into the QCA and also to reduce the spherical aberration introduced by bullet like silicon lenses [4] at the expense of less efficient beam collimation. A far-field measurement of the QCA emission from the lens facet with no anti-reflection coating at a distance of 0.7 cm was performed, producing a spot size with a diameter of ~1 mm as shown in Fig. 3SI. The SRR/graphene device is placed at a distance of 1 cm from the lens in the experiment so to determine the spot size at this further distance the ray tracing package in COMSOL was used. The model was calibrated to have a spot size of 1 mm at 0.7 cm and the resultant spot size at a distance of 1 cm from the lens is simulated to be 1.2 mm. Device 2 has separate SRR areas which are 1.7 mm x 1.7 mm in size so we can be sure that, when correctly aligned, only 1 of these SRR areas is reflecting back into the QCA at any one time and hence the simulated reflectivities of these individual sections are valid.

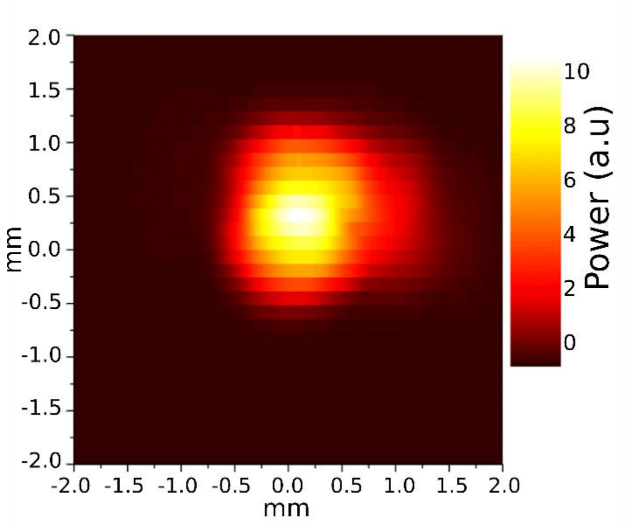

Fig 3SI: Far field spot size measurement from the lens side of the QCA when no anti-reflective coating was used at a distance of 0.7 mm. A Golay cell with aperture of 1 mm is used to measure the output power at different lateral positions.

4. Feedback with graphene device

In order to highlight the importance of implementing SRR resonant arrays in these devices a control device is fabricated in the same way with no SRRs deposited onto the graphene area. This device was then used as a tunable mirror in the external QCA cavity with the main reflection coming from the back of the sample coated with silver paste with the graphene layer acting as a variable THz absorber [5]. The absorption of the graphene will increase as the conductivity increases from 0.05 to 0.11 mS, tuned by varying the back-gate voltage. In Fig. 4SI the absorption is low when the back-gate voltage is at V_{Dirac} resulting in a minimum in absorption and therefore a maximum in feedback power. This results in a maximum in the output power of the QCA. As the voltage is moved away from V_{Dirac}, the absorption of the graphene increases and the QCA output power reduces. The graphene device is modulating the output power, however despite the laser operating at the sensitive region around the threshold current there is only a small modulation effect for large voltage sweeps of around 200 V. The SRR/graphene device can achieve much greater modulation depths with smaller voltage sweeps and also allows for the lithographic tuning of the dispersion which is not possible with a pure graphene device.

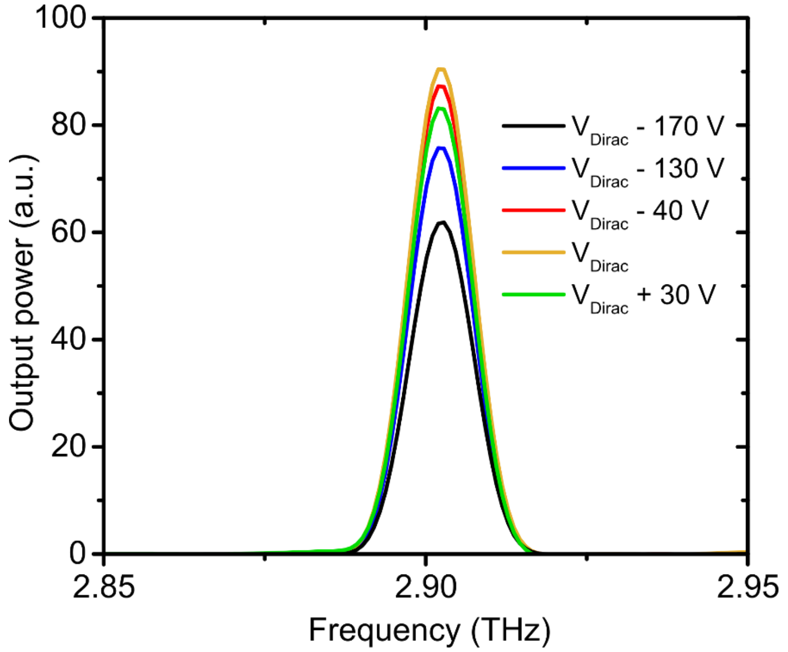

Fig 4SI: FTIR measurement of output power vs frequency of the QCA (running at current I=880 mA) when the graphene device is used as an external cavity mirror with different voltages applied to the device back-gate.

References

1. Yu PY, Cardona M. Fundamentals of Semiconductors: Physics and Materials Properties. *Springer* 2010: 296-297
2. Lee SH, Choi M, Kim TT, Lee S, Liu M, Yin X, Choi HK, Lee SS, Choi CG, Choi SY, Zhang X. Switching terahertz waves with gate-controlled active graphene metamaterials. *Nat Methods* 2012; **11**: 936-941.
3. Ordal MA., Bell RJ, Alexander RW, Long LL, Querry MR. Optical properties of fourteen metals in the infrared and far infrared: Al, Co, Cu, Au, Fe, Pb, Mo, Ni, Pd, Pt, Ag, Ti, V, and W. *Appl Optics* 1985; **24**: 4493-4499.
4. Degl'Innocenti R, Shah YD, Jessop DS, Ren Y, Mitrofanov O, Beere HE, Ritchie DA. Hollow metallic waveguides integrated with terahertz quantum cascade lasers. *Opt Express* 2014; **22**: 24439-24449.
5. Sensale-Rodriguez B, Yan R, Kelly MM, Fang T, Tahy K, Hwang WS, Jena D, Liu L, Xing HG. Broadband graphene terahertz modulators enabled by intraband transitions. *Nat Commun* 2012; **3**: 780.
